# A Practical Approach to Managing Patients With Myasthenia Gravis—Opinions and a Review of the Literature

**DOI:** 10.3389/fneur.2020.00604

**Published:** 2020-07-07

**Authors:** Maria Elena Farrugia, John A. Goodfellow

**Affiliations:** ^1^Neurology Department, Institute of Neurological Sciences, Queen Elizabeth University Hospital, Glasgow, United Kingdom; ^2^Neuroimmunology Laboratory, Laboratory Medicine and Facilities Building, Queen Elizabeth University Hospital, Glasgow, United Kingdom

**Keywords:** ocular myasthenia, generalized myasthenia, refractory myasthenia, thymectomy, myasthenic crisis, fatigue, dysfunctional breathing

## Abstract

When the diagnosis of myasthenia gravis (MG) has been secured, the aim of management should be prompt symptom control and the induction of remission or minimal manifestations. Symptom control, with acetylcholinesterase inhibitors such as pyridostigmine, is commonly employed. This may be sufficient in mild disease. There is no single universally accepted treatment regimen. Corticosteroids are the mainstay of immunosuppressive treatment in patients with more than mild MG to induce remission. Immunosuppressive therapies, such as azathioprine are prescribed in addition to but sometimes instead of corticosteroids when background comorbidities preclude or restrict the use of steroids. Rituximab has a role in refractory MG, while plasmapheresis and immunoglobulin therapy are commonly prescribed to treat MG crisis and in some cases of refractory MG. Data from the MGTX trial showed clear evidence that thymectomy is beneficial in patients with acetylcholine receptor (AChR) antibody positive generalized MG, up to the age of 65 years. Minimally invasive thymectomy surgery including robotic-assisted thymectomy surgery has further revolutionized thymectomy and the management of MG. Ocular MG is not life-threatening but can be significantly disabling when diplopia is persistent. There is evidence to support early treatment with corticosteroids when ocular motility is abnormal and fails to respond to symptomatic treatment. Treatment needs to be individualized in the older age-group depending on specific comorbidities. In the younger age-groups, particularly in women, consideration must be given to the potential teratogenicity of certain therapies. Novel therapies are being developed and trialed, including ones that inhibit complement-induced immunological pathways or interfere with antibody-recycling pathways. Fatigue is common in MG and should be duly identified from fatigable weakness and managed with a combination of physical therapy with or without psychological support. MG patients may also develop dysfunctional breathing and the necessary respiratory physiotherapy techniques need to be implemented to alleviate the patient's symptoms of dyspnoea. In this review, we discuss various facets of myasthenia management in adults with ocular and generalized disease, including some practical approaches and our personal opinions based on our experience.

## Introduction

Myasthenia gravis (MG) is a rare acquired autoimmune disorder of the neuromuscular junction (NMJ), caused by antibodies that target the post-synaptic membrane (1). These antibodies commonly are to the nicotinic acetylcholine receptor (AChR) but in a smaller proportion of cases, antibodies to muscle specific tyrosine kinase (MuSK) or to lipoprotein receptor-related protein 4 (Lrp-4) can be present instead (1–3). In an even smaller cohort of MG patients, no antibodies are detected on conventional antibody assay testing and we refer to these patients as “seronegative.” Patients with MG typically present with fatigable muscle weakness. They commonly present first with ocular manifestations such as asymmetrical fatigable ptosis with or without blurred or double vision. The majority, however, evolve further into generalized muscle weakness involving the facial and bulbar muscles, the neck and axial muscles and the limbs, with the upper limbs often being more severely affected than the lower limbs. In myasthenic crisis, the severe end of the disease spectrum, there is neuromuscular dysphagia rapidly evolving into complete loss of swallow function, and often in association with respiratory muscle weakness and type 2 respiratory failure. This is a clinical emergency that requires management in an intensive care setting. Therapies in the field of MG have significantly advanced over the years. Now, more than ever, the treating physician must carefully contemplate which treatments are best suited for an individual MG patient since the “one size fits all” approach may not be as relevant. There are specific clinical scenarios where one must be extra cautious, for instance the newly diagnosed young female patient, who may be imminently planning a pregnancy, in contrast to a newly diagnosed elderly patient with multiple comorbidities. This review discusses the literature with some emphasis on our practice based over a time-span of over a decade where we have treated an excess of 900 MG patients.

## Pharmacological Therapies in Generalized MG

Medical therapies are used in MG patients for either direct alleviation of symptoms, or as immunomodulatory drugs with the aim of dampening the underlying immunopathology causing the disease. The aim of treatment is to induce remission (pharmacological in the majority or complete stable remission which is rarely achieved) or minimal manifestations (MM). The Myasthenia gravis Foundation of America (MGFA) post-intervention status (PIS) (4) defines MM in a patient who has no symptoms or functional limitations from MG but has some weakness on examination of some muscles. There are four different categories of MM depending on whether the patient is receiving treatment and if this includes immunosuppression and/or symptomatic treatment (for example pyridostigmine as will be discussed below). This contrasts to complete stable remission (CSR) where the patient has no symptoms of MG and no weakness (excluding residual weakness of eye closure) and has received no therapy for a minimum period of 1 year, and pharmacological remission (PR) which is the same as CSR but the patient would have received some therapy for MG excluding symptomatic treatment.

## Symptomatic Therapies

Pyridostigmine is by far the most commonly used symptomatic therapy. This is an acetylcholinesterase inhibitor which blocks the degradation of acetylcholine at peripheral cholinergic synapses, including the neuromuscular junction (NMJ). Originally, physostigmine and prostigmine (neostigmine) were identified by Mary Broadfoot Walker, a physician in Scotland in the late 1880s, as drugs that temporarily improved muscle strength in patients with MG (5). These drugs work by prolonging the action of any acetylcholine released into the synaptic cleft and compensates for the structural and functional deficits in NMJ transmission that characterizes MG. In early or mild disease pyridostigmine allows significant and rapid improvement in muscle strength (6, 7). However, with longstanding or severe disease this pharmacological compensation may be insufficient and there may be minimal clinical effect. Peak blood levels of pyridostigmine occur 1.5–3 h after oral intake but significant clinical effect occurs within 30 min. Dosing 4–5 times per day leads to very stable blood levels. Renal impairment leads to reduced clearance of pyridostigmine and doses must be adjusted.

Patients are usually prescribed doses of 180–240 mg daily but patients may require up to 480 mg daily. Although generally well-tolerated, side effects from pyridostigmine are very common, are usually dose dependent, and can be debilitating necessitating reduction of dose or slower titration. Most side effects arise from the action of pyridostigmine at non-NMJ muscarinic peripheral synapses and include, gastrointestinal disturbance (abdominal cramps, bloating, diarrhea, frequency, nausea), urinary frequency, hypotension, bradycardia, sweating, salivation, lacrimation, increased bronchial secretions, and other symptoms of cholinergic excess. Some elderly patients can be extremely sensitive to the cardiac side effects and have experienced syncope even with low doses of pyridostigmine. Some asthmatic patients may show increased sensitivity and experience increased bronchospasm with pyridostigmine. At high doses side effects can be severe, and lead to the entity of “cholinergic crisis,” where neuromuscular weakness worsens along with the above symptoms leading to bulbar or respiratory crisis from drug excess rather than worsening MG (8). Such extreme manifestations are uncommon but it is very frequent for patients to have gastrointestinal symptoms on starting or increasing doses. These tend to lessen within a few days but can persist in some. Propantheline is an antimuscarinic agent that counteracts many of the cholinergic side effects of pyridostigmine without reducing its action at the NMJ. It can be very effective at reducing the side effects of pyridostigmine if given ~15 min beforehand. Loperamide can alternatively be prescribed but is not as effective at reducing the other muscarinic side effects. When patients fail to respond to pyridostigmine, the physician should be cautious about increasing the dose particularly in dysphagic patients, since pyridostimgine will increase salivary secretions and exacerbate their swallowing difficulties.

Neostigmine is an alternative acetylcholine esterase inhibitor that can be used in MG (9, 10). This should only be given via the subcutaneous route in MG and not intravenously. It is useful in patients with MG who cannot absorb via the oral route (e.g., a MG patient with acute bowel obstruction) but should not be first line if the patient has impaired swallow. Swallowing difficulties are very common in patients with MG and if there are concerns about aspiration with oral intake, including medications, the first strategy should always be to place a nasogastric tube and administer pyridostigmine via this. Only if this cannot be undertaken should subcutaneous neostigmine be used. It has the same side effect profile as pyridostigmine albeit with more marked cardioinhibitory effects and a shorter half-life leading to more frequent dosing. However, neostigmine should always be used with caution since it may cause excessive salivary secretions and as a result may further negatively impact and exacerbate swallowing difficulties.

Experimental models of AChR deficiency show how oral ß-2 adrenergic receptor agonists such as salbutamol enhance function of the NMJ (11). Oral salbutamol can rarely be of clinical utility in mild autoimmune MG disease too especially where the patient has not tolerated pyridostigmine. We have used successfully in a couple of patients. Side effects commonly include tachycardia, tremor and a sense of anxiety and these can be limiting factors. MG patients with MuSK antibodies tolerate albuterol and 3,4-diaminopyridine (12) more than pyridostigmine which, in MuSK-MG, is commonly associated with enhanced side effects especially of cramp and muscle fasciculations. A small clinical trial (phase IIB) studying amifampridine phosphate in MuSK-MG demonstrated this drug to be safe and effective (13). Ephedrine, a sympathomimetic agent, can also be used as an add-on treatment and improves symptoms and weakness (14). Tirasemtiv has been explored in a clinical trial and found to increase the muscle response to calcium and improves muscle strength in MG (15). This remains an experimental drug.

## Immunomodulatory Therapies for Generalized MG

### Corticosteroids

Prednisolone or prednisone constitute the main immunomodulatory therapy in the long-term management of patients with MG (16, 17). The majority will require long-term oral corticosteroid therapy and it is crucial to have the appropriate discussion with newly diagnosed patients, indicating that this will not be a short course of treatment. It is equally important to discuss with patients the long list of potential side effects from steroids, necessitating bone and gastric protection. Patients should also be adequately monitored for the development of diabetes mellitus, hypertension, with careful counseling on potential excessive weight gain and the necessary dietary changes that they may need to pre-emptively and pro-actively address. Other side effects include the formation of cataracts, raised intraocular pressures, mood and sleep disturbances, peripheral oedema and susceptibility to frequent infections or even sepsis. The latter may result in failure of response to conventional MG therapies or even a chronic refractory state and decline in status with multiple hospital admissions.

There can be a paradoxical worsening of MG symptoms on commencing corticosteroids at high doses (18). Therefore, our practice is to start at a low dose and escalate the dose gradually (16). Our initial practice was to use an alternate day regimen of steroids, where side effects are probably reduced when compared to the daily dosing schedule. However, we have encountered many difficulties with the alternate day regimen including patients and physicians in primary and secondary care becoming easily confused, and we have therefore resorted, in the last 3 years or so, to applying the daily steroid regimen. We initiate prednisolone at 5 mg daily and increase every third dose (day) by 5 mg until we achieve stability in MG symptoms and significant improvement, with our ceiling dose usually being 50 mg daily but higher doses have been prescribed in a few select cases.

We treat the majority of patients in the outpatient setting, giving clear instructions to the primary care physician and to the patient, with contact details of the myasthenia team. The nurse specialist phones in on the patient regularly to ensure that the treatment plan is being ensued and to monitor patients' symptoms over the phone. In patients demonstrating significant bulbar weakness, our preference is to admit them immediately to the neurology ward and to initiate treatment accordingly including symptomatic treatment with pyridostigmine and where necessary intravenous immunoglobulin (ivIG).

With the slow steroid dose escalation that we apply, patients improve after 2–4 months of initiation, but some do take much longer to improve significantly. This can be problematic in some, and occurs in circa 20% of patients that we manage. In patients with moderate bulbar muscle involvement or disabling fatigable limb weakness, we prefer to admit to the acute neurology ward or to the day-case ward (if they are generally stable) to treat them with a course of ivIG during the steroid escalation process in order to help expedite the process of their recovery. Occasionally, patients require more than a single course of ivIG to help stabilize their symptoms or to significantly improve their symptoms while increasing their corticosteroid dose. Some patients may not respond to ivIG. In this case, we employ plasma exchange (PE) if we feel their symptoms are sufficiently disabling. If patients are stable (but symptomatic) then PE can be administered in a day-case unit in an outpatient setting and PE carried out through peripheral venous access.

The slow steroid escalation regimen of treatment that we employ is in contrast to the early fast-acting treatment (EFT) strategies applied by the Japanese group (19, 20). This strategy always involves patients being admitted to hospital for treatment where they would receive 1–2 plasmapheresis sessions followed immediately by high-dose intravenous methylprednisolone (0.5–1 g), with or without intravenous immunoglobulin therapy. Treatment would be repeated if significant improvement did not take place. Patients were then discharged from hospital on the lowest dose possible of oral steroids. In some patients, who did not have severe MG symptoms, high dose methylprednisolone was not required. Achievement of MM was more frequent and occurred earlier in the EFT therapy cohort were compared to those in the non-EFT one (19, 20). While this regimen of treatment is highly attractive, it does require easy access to neurology inpatient beds and the necessary manpower (for instance accessibility to the plasmapheresis team) and would not be practical in our regional neurology center (which has 21 neurology beds serving a population of 2 million).

### Steroid Sparing Immunosuppressive Agents

Until recently our practice has been to initiate a steroid sparing agent such as azathioprine, almost simultaneously as initiating corticosteroids and using a dose of 2.5 mg/kg/day (17). This was based on the study by Palace et al. (21) which showed that azathioprine was an effective adjunct treatment to prednisolone and was effective in reducing the long-term maintenance prednisolone dose, in reducing relapses, and in achieving remission in the long-term. However, our practice changed a few years ago (16, 22), when we began to treat newly-diagnosed MG patients with steroids alone first. A steroid-sparing immunosuppressive agent would be later added if the patient relapsed while reducing their steroid dose indicating that they will require more than 10 mg daily of prednisolone to maintain MM and thus justifying the addition of such an agent. We also consider adding in immunosuppression early if the patient has pre-existing comorbidities such as diabetes, significant depression (with steroids potentially exacerbating their mood), osteoporosis, leg ulcerations, that would be compounded by several-month treatment with corticosteroids. Also, in patients who are demonstrating a slow response with corticosteroid treatment then we would an immunosuppressant early in the course of treatment. Furthermore, in some patients, corticosteroid treatment is absolutely or relatively contraindicated because of background comorbidities and in this scenario we immediately prescribe a steroid-sparing immunosuppressant agent without the addition of steroids. Stabilization can be prolonged with this strategy, and we prescribe ivIG in the interim with or without low-dose corticosteroids depending on the clinical picture. Some patients refuse to be started on steroids because of concerns of side effects and in these circumstances adding a steroid-sparing immunosuppressant at diagnosis is a viable option. A retrospective study by Abuzinadah et al. (23), showed that a satisfactory response (which included CSR, PR, and MM) was achieved in about 50% of MG patients with generalized disease when they were maintained on low dose prednisolone, without a steroid-sparing immunosuppressant with follow-up extending up to 6 years.

We advocate checking thiopurine S-methyltransferase (TPMT) levels (24) prior to initiating azathioprine treatment. If levels are in the normal range, we initiate azathioprine at 25 mg daily and increase weekly by 25 mg until target dose is reached, with blood monitoring carried out in primary practice. Generally, the drug is well-tolerated and we rarely encounter idiosyncratic reactions in our population. The drug however takes 8–12 months to become effective and we counsel patients about this. In our opinion the drug is not entirely benign and we have observed many patients develop multiple skin lesions namely actinic keratosis, as a result of long-term azathioprine use and also skin malignancies such as squamous cell carcinoma. If the TPMT levels are deficient but not absent, then we consider using lower doses of azathioprine, monitoring the level of the active metabolite, 6-thioguanine nucleotides (6-TGN), in the blood and titrating the dose accordingly.

Our second steroid-sparing agent of choice is mycophenolate mofetil (MMF) at a dose of 1 g twice daily. In general, we have found it practical to use this drug and it is well-tolerated and (as previously reported in the literature) (25, 26) except for a small number of patients who complain of associated side effects including disabling dizziness and insomnia, and have discontinued this as a result. In patients with very high body mass indices, we have used doses of up to 2.5 g daily. Infrequently we have prescribed mycophenolic acid which can be better tolerated than MMF, in those with side effects from MMF. We find that the efficacy of MMF is noted after circa 6 months of treatment as was also observed in previous studies (27). Based on our clinical observations, and in contrast to the findings from a previous randomized controlled trial (28) oral weekly methotrexate is as effective as MMF and its efficacy becomes apparent at around the same time-point as MMF. It is about 20 times cheaper than MMF. Nausea and vomiting can be limiting side effects experienced by some. In general, folic acid 5 mg daily is prescribed day 4 after methotrexate but when nausea is prominent, daily folic acid (except for the day of methotrexate dosing) can help alleviate this. Ciclosporin (used at a dose of 3.5 mg/kg/day) is probably the most potent immunosuppressive agent with the added advantage that it is not teratogenic (29). From our clinical observations, we have deduced that ciclosporin is, at minimum, effective within 3 months of initiation. However, we have observed that the majority of patients prescribed this drug run into problems with significant side effects including hypertension, alteration in their glomerular filtration rates, nephrotoxicity, tremor and in female patients also problems with hirsutism. We have prescribed ciclosporin in around 25 MG patients, where they have proven refractory to other steroid-sparing immunosuppressants, and usually belonging to a younger age-group. We avoid prescribing in older patients because of the potential complications and side effects and aim to reserve for younger patient groups. Tacrolimus is of similar efficacy (30) but with a similar side effect profile as ciclosporin. We have not prescribed cyclophosphamide in MG but there is a role for prescribing this drug as a monthly intravenous pulsed treatment in patients with refractory disease and who are unable to reduce their maintenance steroid doses (31), and this is generally tolerated without significant side effects.

### Withdrawing Symptomatic Therapies and Achieving Maintenance Therapy

When the MG status starts to stabilize, MG patients no longer experience the significant fluctuation and variability in symptoms, become less fatigable and their strength starts to normalize. We educate patients about this time-point being reached and trying to recognize when they no longer need to reach out for their next pyridostigmine dose which is a good prognostic sign for stabilization. At that stage, while maintaining the same dose of corticosteroids, we advise patients to reduce their pyridostigmine dose by 30 mg per week (or sometimes faster), with the aim to wean this altogether but in some cases patients prefer or require to remain on low doses of up to 120 mg daily. Reduction of their steroid dose then ensues following a similar regimen previously described (16) −5 mg reduction per month down to 20 mg daily, then 2.5 mg reductions per month down to 10 mg daily, then 1 mg reduction per month or slower, aiming to reach 5 mg daily. In some cases, it is possible to wean steroids altogether especially if a steroid sparing immunosuppressive agent has already been added. However, if this is not the case then careful consideration needs to be taken, with detailed discussion with the patient, about withdrawing steroids altogether and a potential risk of future relapse. There is an argument for maintaining on low-dose prednisolone such as 5 mg daily for life where the cumulative life-time risk is likely to be small vs. further reduction or absolute withdrawal of prednisolone that might trigger a significant relapse of MG. In our experience, most patients favor the former option. Also, we are of the opinion that the long-term risk of such low-dose prednisolone (development of diabetes, hypertension, osteoporosis, glaucoma etc.) is significantly less than for example being maintained on 100 mg of azathioprine for life—although there are no long-term studies that quantitate this risk.

## Reducing the Dose of Second-Line Agents

When patients have achieved pharmacological remission and have successfully withdrawn corticosteroids, then it would be sensible to consider a gentle reduction in their steroid sparing immunosuppressant dose (17). The difficulties are 2-fold: firstly there is little data on the actual risk of relapse on withdrawal of immunosuppression and secondly there is no consensus or guideline on how rapidly the dose should be reduced. With regards to the first point, the limited studies on this indicate that the risk of relapse on withdrawal of immunosuppression may be rather high. In two respective studies, more than 50% of patients who were in CSR and who were prescribed azathioprine (32) and nearly all patients who had significantly reduced the dose or withdrawn MMF, experienced a relapse in their MG (33) necessitating the reintroduction of immunosuppression. With regards to the second difficulty: we usually take an ultra-conservative approach when reducing the dose of any immunsuppressant. In the case of azathioprine we reduce the dose by 25 mg every 6 months (infrequently weaning altogether) while with MMF we reduce no faster by 500 mg per year, as previously reported (34). We always advocate close monitoring of patients' MG status and symptoms during the reduction process. The rate of CSR is low and we often opt, after discussion with patients and balancing the decision against their age and comorbidities, to maintain them on the lowest dose possible of immunosuppressant in the long-term unless there is a pressing requirement that this is discontinued altogether.

### Thymectomy

Thymectomy in generalized AChR antibody positive MG should be considered as early as possible in the management plan and thymectomy should be performed where relevant when the MG status has been stabilized (17). Imaging of the thymus gland, using CT or MR modalities, should be performed in all AChR antibody positive MG patients, also to rule out thymoma and in the younger patients to look for evidence thymic hyperplasia. The role of the thymus gland in driving MG has been known for almost a century (35, 36). The results from the international thymectomy trial (MGTX) have been crucial in underscoring the role of thymectomy in the management of MG (37). In this trial, non-thymoma MG patients up to the age of 65, with generalized disease and with positive AChR antibodies, were recruited. Patients whose MG onset was up to 5 years prior were recruited. The goal of the surgical procedure, in those who received thymectomy, was to remove all thymic tissue including ectopic tissue and surrounding fat. The results showed that patients who had thymectomy (which involved an extended trans-sternal procedure) required lesser doses of corticosteroids both in the short and in the long-term (38), had better functional outcomes, were less likely to be hospitalized due to their MG and were less likely to require additional immunosuppression with azathioprine for instance. The benefit was seen across all age-groups and was sustained on follow-up. This trial has been pivotal in the way we neurologists are now approaching MG management. Thymectomy now is more widely offered to patients with generalized disease associated with AChR antibodies, including patients with late-onset MG and up to the age of 65, as part of the overall treatment of their MG.

Minimally-invasive thymectomy surgery has been further revolutionary in the field. Reports of video-assisted thorascopic surgery (VATS) thymectomy began to emerge in 1993 and 1994, with a number of centers using alone or in combination with a trans-cervical approach (39–41). Reports of robotic surgery in the field of thoracic surgery began to emerge in the early 2000s (42) with the use of the da Vinci robotic system applied in a 28-year old patient with MG. With both types of minimally invasive procedures, patients are reported to experience less blood loss intra-operatively, complain of less pain post-operatively and have a shorter post-operative hospital stay when compared to open thymectomy. In a systemic review comparing robotic assisted thymectomy surgery (RATS) with VATS and open surgery (43), there are clear advantages of RATS or VATS over open surgery, but no significant advantage of RATS over VATS at least to date. Clinical outcomes have been compared in a retrospective study in MG and found to be comparable between thoracoscopic vs. trans-sternal thymecetomy (44). Data analyses, after propensity score matching, also confirmed that robotic thymectomy in early stage thymoma was safe and feasible with oncological outcomes that were comparable to trans-sternal thymectomy (45) and in thymoma exceeding 5 cm (46, 47). In relation to MG outcomes, it would be very challenging to design a further trial that would compare clinical MG status and outcomes after open thymectomy vs. minimally invasive surgery.

In our experience, patients with non-thymoma MG are now more encouraged to pursue thymectomy, during the course of their MG management, when provided with the results from the MGTX trial. We have also observed that patients are also more comfortable in pursuing minimally invasive thymectomy surgery in contrast to open surgical approaches. For the past 3 years, our thoracic surgeons have been employing RATS, which we perceive as further advantageous specifically from the perspective of post-operative morbidity. For those who are in employment, who drive, who are parents looking after young children, and also for those younger adults who may be pursuing studies at school or university, RATS evokes less anxiety about the post-operative period impacting on their work, studies, social, or family life. Minimally invasive thymectomy procedures also overcome the aesthetic problems that patients faced with open thymectomy mediastinal scars. We, as a center, have also gained confidence in referring older MG patients for thymectomy, acknowledging that there is data to support its benefit also in this age-group (48, 49), and since the MGTX trial, we have been consistently referring patients up to the age of 65.

Although it is perceived that that there is a 2-year window of opportunity for thymectomy from disease onset, there is no evidence to suggest that the MG status is negatively impacted when thymectomy is performed beyond this time-frame. In the MGTX trial there was no evidence to support that patients who had thymectomy within 2 years did better than those who had thymectomy within 5 years of disease onset. This is particularly relevant to patients, who have proven refractory to all conventional immunosuppression, and where thymectomy at a later time-point in their disease could potentially offer additional benefit; we have been exploring this as an option in a small category of patients. In contrast, there are various reports indicating that thymectomy is contraindicated in MuSK-MG, with patients' MG status often worsening after the procedure and, therefore, thymectomy should not be pursued if MuSK antibodies are present (50). The jury is out as to whether thymectomy would benefit MG patients without AChR or MuSK antibodies (traditionally referred to as double seronegative) and if there is a role for thymectomy in MG with LRP-4 antibodies (51). Leite et al. (52) had shown that the thymic abnormalities in double seronegative patients had more thymic abnormalities than the MuSK-MG thymus, but less than seen in the AChR-MG cohort (52). Thus, these patients may benefit from thymectomy too but this is an area that requires further research.

Thymoma, in contrast, is a rare epithelial tumor of the anterior mediastinum and 50% of cases occur in association with MG. Thymoma occurring in association with MG, should always be surgically removed (17). Minimally invasive surgical approaches are feasible in most but may not be possible in the larger tumors. Complete surgical resection is aimed for but radiotherapy may be required for invasive thymomas. Where resection is incomplete and/or surgical margins are positive for thymoma, radiotherapy improves the prognosis by 50–60% (53, 54). Thymomas are also chemosensitive. Platinum-based agents show consistent efficacy (55) and can improve the outcome of Masaoka stage III and IV thymomas or recurrent thymomas. Non-platinum based regimens are also prescribed in some tumors and the role of immunotherapy still remains to be further investigated.

### Ocular MG

Isolated ocular myasthenia is rare. While ptosis and diplopia are common presenting symptoms including in patients who will eventually evolve into generalized myasthenia, only 20% of patients will turn out to have pure ocular MG—signifying that these patients will never develop generalized disease) (56, 57). The diagnostic difficulty with this entity is that only 50% have detectable antibodies to the AChR (57). Single fiber EMG studies support the diagnosis of neuromuscular transmission failure in patients without detectable antibodies, including ocular myasthenia (58). The main differential diagnoses include thyroid eye disease, and a progressive external ophthalmoplegia associated with a mitochondrial disorder. The latter group of patients may also have some minor abnormalities on single fiber EMG studies with borderline increased jitter values making the diagnosis even more challenging (58). Other diagnostic cues are therefore crucial, and ultimately a muscle biopsy may be necessary to clinch the diagnosis.

### First-Line Pharmacological Therapy in Ocular MG

While ocular MG is not life-threatening, diplopia is a very disabling symptom. It significantly impacts an individual's quality of life—it impacts patients' driving ability, it may impact their employment, their social life, and their hobbies including sports, reading etc. When a patient presents with ocular myasthenia, the first treatment that should be initiated is pyridostigmine in order to achieve symptom control and to determine reversibility. This may be sufficient in patients with mild symptoms and signs, but is unlikely to be adequate in patients with significant ocular motility disturbance. If patients remain symptomatic despite maximal doses of pyridostigmine, then the next step should be prompt treatment with corticosteroids (59–63). Early treatment of ocular myasthenia improves the chances of reversibility or significant improvement in the long-term (64). There is some evidence to indicate that early treatment of ocular myasthenia delays or prevents the development of generalized disease (64–66). Delaying corticosteroid treatment, in our experience, reduces the chances of recovery of the extraocular muscles. In some patients, in spite of prompt and adequate treatment, they do not respond to therapies and are left with a fixed ophthalmoplegia in the absence of any other signs or symptoms. This may reflect the complex sarcomeric organization (67, 68), gene expression (69), distinct complement expression (68, 70), and unique metabolic demands and vulnerability of mitochondrial oxidation pathways within the extraocular muscles (71) that are susceptible to disease including autoimmune disorders. In patients who are refractory to treatment, and especially when they have no detectable antibodies and/or equivocal SFEMG findings, there is scope for investigating with an MRI scan of the orbits with gadolinium to exclude alternative, namely inflammatory, processes for instance thyroiditis. Commonly in ocular myasthenia patients with refractory disease and fixed ophthalmoplegia, the MRI shows atrophic extraocular muscles with asymmetric involvement and with no enhancement following gadolinium administration.

The ceiling steroid dose in ocular myasthenia is deemed to be lower than that used in generalized myasthenia (16, 57). One usually aims for a maximal steroid dose (prednisolone/prednisone) of around 25 mg daily (or equivalent of 50 mg alternate days) but in some instances higher doses may need to be considered particularly if there is a delay in the correction of the ocular motility disturbance and if diplopia remains a persistent symptom. Recovery of the extraocular muscles in ocular myasthenia may take several months and there may be scope for adding in immunosuppressive agents for the same reasons as in generalized MG (16, 22, 72). The indications for this includes patients whose ocular motility does not respond to corticosteroids alone, or who experience frequent relapses and are unable to reduce the corticosteroid dose below an acceptable level, or the physician feels that additional treatment is required especially if there has been incomplete response to corticosteroids and pyridostigmine. Other patients are unable to tolerate corticosteroids or may have comorbidities such as diabetes, osteoporosis, depression, or glaucoma that preclude the long-term use of steroids.

It is important to monitor patient's response to treatment carefully and working with an orthoptist can be of immense assistance. There also needs to be an objective assessment of ptosis and ocular motility for instance using the Jampolsky scheme (73, 74), and collaborative work with an orthoptist is often very helpful in monitoring response to treatment and progress.

### Non-pharmacological Therapies in Ocular MG

In the short term, patients may be fitted with a Fresnel prism to allow some correction of their double vision (75). Reducing the strength of the prism over time is a clear indication of response and improvement. Some patients, however, will continue to rely on their prism in the long-term. Using a patch over one eye in the short-term is another option for some patients to help obliterate the false image while others tolerate using an occlusive contact lens.

Residual ptosis can be a significant problem in some patients either causing obstruction of one's vision or from an aesthetic perspective. In older patients, ptosis may be aggravated further by senile dehiscence or dermatochalasia. In general, if patients' ptosis does not reverse in spite of maximal treatment received over a 2-year period, then the chances of recovery after that period of time are rather slim. In a select group of patients, ptosis repair surgery performed by an oculoplastic surgeon may be indicated (72, 76). The surgeon needs to ensure that the risk of corneal exposure is minimal and repeated procedures are best to be avoided. In contrast using ptosis props is a less invasive way of dealing with the problem but some patients complain that these cause discomfort or corneal dryness since the props limit blinking, and may be simply impractical for some. In some patients, the extraocular motility may remain abnormal in spite of adequate treatment with steroids, and may become fixed. In a highly select group, strabismus surgery may be of benefit but careful discussion with an ophthalmologist who specializes in squint surgery is required for these cases. Botulinum toxin to correct the strabismus should be avoided altogether in MG because of the toxin's systemic effects, which may destabilize MG patients even when their status (other than their ocular features) has been stable for many years (77).

Thymectomy in ocular myasthenia remains controversial but there are various small studies indicating that this is beneficial particularly when considered early in the disease (78–82). The task force for the EFNS/ENS guidelines (62) agreed that thymectomy is not recommended for ocular myasthenia as first-line treatment but should be considered if drug therapy was not successful and may prevent MG generalization (good practice point). Given that ocular myasthenia often evolves into generalized disease (and there are no markers to predict this) and given the increased access to minimally invasive thymectomy surgery, early intervention may be of benefit. For these reasons, we have increasingly been referring ocular MG patients for thymectomy in the last 3 years. Furthermore, it is unknown, if early thymectomy may also prevent these patients developing a fixed ophthalmoplegia in the long-term.

## MG in Specific Patient Groups

### The Pregnant Patient

In practice, the majority of MG patients, who are treated adequately before pregnancy, do not experience any complications during pregnancy or in the post-partum phase. However, some report an increased risk of MG relapse during pregnancy that varies between 17% (83) to 41% (84). Some patients' MG status improves during pregnancy (85) as one observes with other autoimmune conditions such as multiple sclerosis. In the ideal scenario, the pregnancy is planned to allow optimization of MG status and withdrawal of teratogenic medications where relevant. Pyridostigmine, corticosteroids, and azathioprine are all safe to be used in pregnancy and should not be discontinued during pregnancy (85, 86). MMF and Methotrexate are teratogenic and should be avoided (85). Ciclosporin and tacrolimus are not teratogenic but their use can be associated with the development of hypertension and gestational diabetes and, therefore, the patient requires close monitoring (85). IvIG and PE are also safe to be used during pregnancy (87). There are some reports suggesting that MG patients are at risk of preterm rupture of membranes (88, 89). We have not encountered this in our practice, however.

Therapy for MG should be optimized where possible before and during pregnancy. The neurologist and obstetrician should be in regular dialogue particularly in the third trimester of pregnancy, when plans should be initiated on how the baby should be best delivered. Medications for MG should continue uninterrupted before and throughout labor. Patients should undergo spontaneous vaginal delivery in most cases and epidural labor analgesia should be considered early in patients who are likely to experience fatigue during labor (90). Nitrous oxide is safe to use (85).

Surgical delivery should be considered in those who MG status is poorly controlled and in those patients where muscle weakness is significant or their MG is considered brittle. Ideally this should be planned adequately in advance with multidisciplinary team discussions throughout but especially in the latter part of the pregnancy. MG patients are usually extremely sensitive to depolarizing muscle relaxants, and should be administered the least possible dose (85). Magnesium sulfate for the treatment of eclampsia should be avoided in MG since this will exacerbate myasthenic weakness (85). Opiates for pain relief especially in the post-partum phase should be used with caution since they too may exacerbate weakness. Breast feeding of the newborn should be encouraged. Neonatal myasthenia, with temporary and usually mild myasthenic weakness, occurs in 10% of neonates due to transplacental transfer of antibodies (86, 91). It usually resolves spontaneously within 3 weeks of the birth of the infant. Rarely the presentation of the neonate can be more complex, especially if the mother's MG was undertreated during pregnancy, and may require the neonate to be managed in an intensive care setting for a short period. Very rarely, infants of MG mothers are born with mild myopathy and—at the severe end of the spectrum—arthrogryposis multiplex congenita (92). The mothers may in fact be asymptomatic or minimally symptomatic with elevated AChR antibodies and some may be asymptomatic with antibodies specific to the fetal AChR γ subunit (92).

### The MG Patient in Crisis

MG crisis occurs in circa 20% of MG patients who are newly presenting with MG (93, 94). It occurs more frequently in MG patients who are undertreated, or who have newly presented and whose treatment is being slowly escalated but whose presentation has evolved more rapidly than therapy has originally been scheduled for stabilization. Patients develop severe muscle weakness including weakness of the respiratory muscles, commonly preceded by severe bulbar weakness with dysphagia, with or without palatal weakness and nasal escape. In this situation, patients require a nasogastric tube to be inserted to allow medications to be administered and for feeding. This clinical picture must be promptly recognized and the patient requires to be monitored closely in hospital, usually in a high dependency unit setting, since this clinical picture often evolves further with significant respiratory muscle weakness. Arterial blood gases should be checked to identify when type 2 respiratory failure occurs. At the bedside, assessing the patient's respiratory rate and forced vital capacity, and observing whether the patient is using their accessory muscles are all helpful measurements, predictors or cues. If parameters allow then the patient could be treated with non-invasive ventilation (NIV) first but if parameters fail to improve or the patient continues to tire with NIV or is intolerant of this, then treatment must be quickly escalated and the patient must be intubated and mechanically ventilated in an intensive care setting.

The two primary pharmacological therapies to treat MG crisis are ivIG, at a dose of 0.4 g/kg/day for 5 days or PE—usually 4–6 exchanges (17). They are equally effective in the treatment of MG crisis or a significant MG relapse (95). We commonly prescribe ivIG first, unless there are contraindications, and resort to PE as second-line therapy if the patient fails to respond to ivIG. However, if PE is readily available we would recommend using as first-line in the context of MG crisis since it is more rapid in its effect than ivIG. This has been our experience and also previously shown by Qureshi et al. (96). PE is not without risk however. It is more invasive, more labor-intensive and more expensive than ivIG (97). PE should be performed via peripheral venous access, where feasible, but central catheters may be necessary in some which pose additional risks of an infection source if mishandled or if left *in situ* for too long (98). The same dose of ivIG could be administered over a shorter period for example 2–3 days if tolerated by the patient. We prefer to administer over 5 days, especially in patients who are ivIG naïve at least initially, and we consider administering over 2–3 days in subsequent treatments.

Corticosteroids are added or increased simultaneously with ivIG or PE therapy (16). In our practice, we still initiate corticosteroids at low doses but then we escalate the dose more rapidly over 5–7 days, since the steroid dip is likely to be counteracted by the simultaneous use of ivIG or PE. The role of acetylcholinesterase inhibitors is limited in MG crisis. They may exacerbate bronchial secretions and so one should be mindful of identifying the clinical situation when they are likely to be of benefit even to the MG patient in crisis. Some patients may require further courses of PE or ivIG 4–5 weeks after their initial therapy and may relapse even after their initial significant improvement. This is because the effect of corticosteroids may be apparent after 6–8 weeks while the effect of ivIG or PE usually lasts circa 4 weeks.

Weaning from the ventilator should be considered when the patient demonstrates an improvement in vital capacity and is strong enough to transition to spontaneous mode ventilation, which allows the patient to initiate breathing (99). The patient should be observed for fatigability with switch-over to assisted-ventilation when they fatigue. There is concomitant improvement in bulbar and neck muscle strength when respiratory muscle improvement is observed. If their cough remains weak and the patient is struggling to clear their airways secretions, then extubation is likely to be precocious and failure is more likely to occur.

Consideration for thymectomy should be considered where relevant and after the patient has been weaned off ventilation and extubated. Also, they should demonstrate stability in their MG status, have been stepped down to a regular ward and are becoming less dependent for their daily activities of daily living. The prognosis of MG crisis is worse in patients with thymoma. In this group of patients, managing their MG crisis can be challenging and response to therapy may be delayed (93). When their MG status has been stabilized, however, thymectomy should follow on promptly when safe to do so.

### The Older MG Patient

World-wide epidemiological studies confirm that the incidence of MG is increasing among male and female patients who are older than 65 years (100–102) and the prevalence is also rising due to patients living longer (103, 104). Multiple comorbidities often exist in older patients. They are less likely to tolerate the more potent immunosuppressive agents that benefit the younger MG patients (105–107). In older patients, careful consideration needs to be given of the potential impact of corticosteroid treatment on other systems for example the development of diabetes, hypertension, obesity with cardiac strain and heart failure, significant osteoporosis with vulnerability to various fractures. They become more vulnerable to infection including recurrent infections and sometimes resulting in life-threatening sepsis especially when more potent immunosuppressive agents such as MMF or Methotrexate are prescribed. Some older patients suffer recurrent infections when managed with maximal immunosuppression for their MG which in turn results in hospitalization, further deconditioning and a significant delay in recovery from their MG. From our experience, we have noted that in the older perhaps frailer patients it may be safer in the longer term to slightly undertreat their MG rather than aim to induce remission, since prescribing conventional doses of immunosuppression in this age-group often leads to fatal consequences. MG patients are also more vulnerable to developing osteoporosis (108) and the prescribing neurologist needs to be aware of this and monitor closely patients' bone densities since osteoporotic fractures result in significant morbidity, chronic pain and reduced mobility, which may already be compromised in an older patient.

### The Refractory MG Patient and Novel Therapies

About 20% of MG patients are refractory to all conventional treatments. Monoclonal antibody treatments that bind the B lymphocyte membrane protein CD20, such as Rituximab have been increasingly prescribed in this group of patients with successful outcomes. The rationale behind preparations such as Rituximab is that they destroy and deplete pathogenic B cells and decrease AChR antibody production. Rituximab influences the whole spectrum of B cell function including antigen presentation, cytokine production, and T cell stimulation and hence has a role in T cell mediated autoimmune diseases too (109). Studies have demonstrated that clinical improvement even with one cycle of Rituximab is sustained (110, 111) allowing subsequent reduction in steroid doses and in some inducing remission (112). Patients with MuSK-MG respond extremely well to Rituximab and the drug often induces remission without the requirement for subsequent infusions (112, 113). Rituximab has a role in patients presenting aggressively and explosively at onset and who are refractory to all conventional therapies. Brauner et al. (114) demonstrated that clinical outcomes were better in patients who were treated early rather than later with Rituximab. There is scope for considering Rituximab in patients who are in crisis and who are not responding to high dose corticosteroids or ivIG or PE, and when patients demonstrate resistance in weaning off ventilation during the treatment pathway of MG crisis. Caution must be exerted in this scenario, acknowledging that Rituximab will not be effective immediately and may pose an added risk to the patient for developing infection. Rituximab is contraindicated during pregnancy (87).

In our experience, where we have treated a small cohort of 17 MG patients with MuSK-MG, AChR-MG, and MG with no detectable antibodies, the majority of patients improved significantly but remain dependent on immunosuppression (unpublished data). Our single MuSK-MG patient, within this small cohort, responded best to Rituximab although this did not induce complete remission of her disease. In contrast, about a third of MG patients did not respond to Rituximab and their MG status was not altered by this therapy. In general, we have found that the drug is well-tolerated with minimal side effects. However, in two patients we have observed delayed neutropenia developing many months after Rituximab treatment, including one patient whose presentation was complicated by two neutropenic sepsis episodes several months after their Rituximab treatment. This has been observed in other patient groups treated with Rituximab (115–117).

In a large systemic review of 169 MG patients who received Rituximab, remission (PR or CSR) and MM was achieved in 72% of MuSK-MG patients in contrast to 30% of AChR-MG patients, with post-treatment relapses being markedly reduced in the MuSK-MG cohort (118). It is still unclear why MuSK-MG patients respond so well to this drug. It would be crucial for biomarkers to be developed that will allow physicians to predict a patient's response to Rituximab. There is also a similar crucial need for robust trial data for this drug, since the efficacy of Rituximab in AChR-MG is still debatable and the studies that are available may be limited by an element of reporting bias (119). This data will also help physicians counsel patients adequately when embarking on this therapy.

MG treatment can also be addressed by switching off complement pathways and their activation, or by altering the Fc region of the antibody such that less antibodies are available for recycling, more are destroyed and thus unavailable for pathogenic processes. Novel therapies have been developed to address both. The efficacy and safety of the terminal complement inhibitor eculizumab (a humanized monoclonal anti-C5 antibody) in MG has been rigorously studied in the REGAIN trial (120, 121). Improvements were noted in all objective MG-related scores and in the patients' quality of life scores for all those actively treated with eculizumab, and were sustained during the 52-week study period. Patients treated in the placebo arm experienced rapid and sustained improvement in their MG status when switched to open-label eculizumab. The drug also improved fatigue scores which in turn correlated strongly with MG-specific outcome measures (122). However, the response among patients in the REGAIN trial was variable with some improving substantially, some modestly and some patients showing no response whatsoever (123). Eculizumab is now a registered therapy for myasthenia gravis. It remains an expensive drug with costs for one patient's treatment per annum amounting to $500,000. It is unclear whether this drug is cost-effective in MG. A trial of zilucoplan, a subcutaneously self-administered inhibitor of complement component 5, has been recently studied (124). The trial confirmed that zilucoplan was safe and well-tolerated and patients rapidly showed clinical improvement with this drug. The extent of clinical response correlated with the level of complement inhibition such that near-complete inhibition was demonstrated to be superior to submaximal inhibition.

Efgartigimod (also known as ARGX-113) has been trialed in generalized MG in a phase-2 randomized double-blind, placebo-controlled study in 15 centers (125). ARGX-113 is the anti-neonatal Fc receptor immunoglobulin IgG1 fragment. It has been modified to increase its normal affinity for IgGs, thus blocking the formation of disease-causing IgG. Efgartigimod was well-tolerated in this trial. In the 12 patients treated with the active drug, there was a rapid decline in total Ig levels and in AChR titers, which in turn correlated with a clinical improvement of their MG, and this was sustained in the majority.

The proteasome inhibitor, Bortezomib, depletes short-lived and long-lived B cells and is applied in the treatment of multiple myeloma (126) and plasmablastic lymphoma (127). It is likely to have a role in the treatment of refractory MG including MuSK antibody positive MG (128) but the development of a sensorimotor polyneuropathy, a recognized side-effect of this drug, is likely to be a limiting factor.

Questions remain unanswered about the long-term safety, efficacy, and tolerability of these novel therapies (meaning after several years of continuous treatment). It is unclear whether long-term complement inhibition, for instance, would pose increased general infection risks particularly in older age-groups. Determining the category of patients who are likely to benefit from these therapies is crucial. Would these therapies be aimed only for “refractory” and “severe” MG? If so, how do we precisely define these entities? Would drug holidays be considered and if so for how long? It is also less clear how cost-effective these novel therapies are, how the various global health systems would fund these drugs and how the different health insurance companies will cover the costs of these drugs. A detailed cost-utility analysis is required that will allow the diverse health systems to better understand the long-term efficacy of these therapies, how improvements in objective measurements translate into better function for the patient, and how they improve patients' quality of life. It would be imperative to ascertain and quantify the potential socioeconomic gains when using these therapies (do these therapies allow individuals to return to their employment, increase independence and reduce dependence on care-givers?) and the impact on reducing in-patient hospital care (reducing hospital admissions including to intensive care units, the requirement for regular ivIG, or frequency of attend clinic appointments due to stable disease etc.).

### Fatigue in MG

Fatigue is common in all neuromuscular conditions including MG, and around 80% of MG patients will experience significant fatigue at some stage of their disease (129, 130). It is distinct from fatigability and muscle weakness and therefore it is crucial that the physician recognizes this entity since its management does not involve escalation of treatment for MG (131). Fatigue is as disabling to the patient as active muscle weakness, and may negatively impact patients' quality of life, their quality time with their family, their employment status, and their social lives. It contributes to the disease burden but is more difficult to assess or objectively measure in the clinic. Fatigue may be problematic even when MG symptoms have largely settled or when the patient has achieved minimal manifestations.

Fatigue is multifactorial. Primary fatigue occurs when muscle weakness and fatigability are active in MG and has an inherent physical component (132) contributing to fatigue. They also complain of cognitive fatigue which patients often allude to as “brain fog” (133). It is difficult to dissect out primary from secondary fatigue, with the latter occurring for various reasons. Patients with MG, often gain weight primarily due to corticosteroid treatment (134), sleep less efficiently (135), move less and develop muscle stiffness and discomfort (136). They are more likely to become anxious and depressed about their physical limitations and the variability and unpredictability of their symptoms (137). They resort to socializing less, they might discontinue their employment, which in turn may have financial consequences, and do less chores in the house or even become virtually house-bound. O'Connor et al. (138) identified that MG patients were more likely to become sedentary even when asymptomatic. It is unclear whether this is learnt behavior or fatigue-driven or simply part of a vicious cycle. Because MG patients exercise less they become quickly deconditioned and often develop breathlessness that is not secondary to respiratory muscle weakness. Their breathing becomes shallow with a tendency to hyperventilate which develops as a learned pattern and is often misinterpreted as a sign of early MG crisis. Their sleep pattern is less efficient. They may develop obstructive sleep apnoea due to weight gain. They socialize less and this in turn negatively impacts their mood further.

Fatigue is not unique to MG but is also prevalent in other neuromuscular disorders such as different types of muscular dystrophy and myotonic dystrophy (DM1). Patients with facioscapulohumeral muscular dystrophy (FSHD) often complain of fatigue and pain, and hypersomnolence is very common in patients with myotonic dystrophy. Various studies have studied the role of exercise in various neuromuscular studies including MG (139, 140). Other studies have explored using cognitive behavioral therapy in combination with graded exercise in MG, DM1, and FSHD including high intensity training and aerobic exercise which led to functional benefits in patients without evidence of damaging muscle (141–144). In a very small and select group of patients, where fatigue is compounded by pain, anxiety and insomnia, and perhaps with an overlay of their myasthenic symptoms (i.e., true MG coexisting with an aspect of a functional neurological disorder) we have managed them also with psychology input and cognitive behavioral therapy (145).

It is challenging when prescribing exercise to MG patients or indeed to any neuromuscular patient. Different types of exercise are suitable for MG patients at different phases of their MG. Aerobic or high intensity training is not possible when MG patients are very symptomatic. In this situation, stretching exercises such as Tai chi, slow flow yoga or pilates are probably most appropriate with emphasis also on balance maintenance. When MG symptoms stabilize, physical therapy should focus on balance and muscle strengthening but physicians should also enquire specifically about other symptoms including pain, residual fatigue, sleep disturbance and mood problems and address these accordingly.

### Dysfunctional Breathing in Myasthenia Gravis

It has long been observed that breathing patterns and the central ventilator drive can be altered in patients with mild or moderate MG (146). In our practice, we have observed several patients, who we deem stable or in minimal manifestations, complaining of dyspnoea as a residual prominent symptom in spite of them not having any objective evidence of respiratory muscle weakness. A very small proportion, may have had a MG crisis at some stage of their disease, which inevitably raises long-term anxiety levels to the patient and their carer, about the potential severity and sometimes unpredictability of the disease. In some, contributory factors are clear and include deconditioning or weight gain. We have identified, through collaborative work with the local respiratory team, that many of these patients have developed dysfunctional breathing (unpublished observation). Our local respiratory physiotherapist has been working with these patients, employing physiotherapy-based breathing pattern modification interventions. These include relaxation of intercostal muscles, accessory muscles and full utilization of the diaphragm thus helping them to regulate and improve their breathing pattern with good results (unpublished).

Dysfunctional breathing has been studied extensively in poorly controlled asthma (147) because it is common and is associated with significantly poor asthma control and lower quality of life. Evidence-based guidelines recommend breathing retraining interventions as adjuvant treatment in uncontrolled asthma. A multicenter randomized controlled trial is currently underway in Denmark to investigate the effect of breathing retraining on the impact on quality of life in poorly controlled asthmatics (148). In a small study (149), 12 MG patients underwent long-term respiratory muscle endurance training, which resulted in a change in their breathing pattern with prolonged expiration. Interestingly patients reported an improvement in their MG symptoms, in their respiratory symptoms and in their physical fitness. This study proves that normocapnic hyperpnea training is a useful adjuvant therapy in MG.

It is therefore imperative that physicians recognize the entity of dysfunctional breathing in MG patients and refer them on for respiratory-based physiotherapy. This is a crucial adjuvant treatment in MG patients, who complain of dyspnea, and intervention helps their overall MG symptoms, improves their exercise capacity and increases their chances of overall recovery with improved quality of life.

### The End-Result—Our Practice and Comparison With Reported Outcomes

When we set up the myasthenia clinic 13 years ago, we primarily aimed this to be a regional service that manages MG patients residing in the West of Scotland. However, we were subsequently referred MG patients who were refractory to standard therapies and who came from other parts of Scotland. Our patient cohort, served over a 13-year period, is heterogeneous including ocular and generalized MG, spanning all age groups (including patients in their tenth decade), with different antibody status and thymic pathology. About 10% of our cohort is refractory to conventional treatments. Our experience, as previously reported in the literature (150), has been that most patients' MG status evolves within the first 2 years of symptom onset. Broadly, CSR has been achieved in 5–10% of our case-load, PR in 20%, MM in 25%, improvement in 35%. About 10% of our cohort's MG status remains unchanged by our therapeutic interventions. Patients were worsened by therapy in 1–2%, and 1% died from direct complications of their MG. Our rate of PR is comparable to what has been reported in the literature but it is difficult to make direct comparisons since our treatment regime has also evolved over time. Mantegazza et al. (151) reported PR in 24% and CSR in 11%. Beghi et al. (152) reported a higher chance of CSR in patients who were younger and who had a shorter disease duration. These findings were echoed in a further study by the same group almost a decade later (153). Yang et al. (154) reported a CSR rate of 60% in patients who received thymectomy for thymic hyperplasia with younger patients having a higher CSR rate. Given that we have put more MG patients forward for thymectomy in the last 3–4 years, it is likely that this would further influence our remission rates. If we were to categorize our patient cohort according to age-groups, thymus pathology, and thymectomy status this would refine our CSR and PR rates, but we have not carried out that detailed analysis to date.

## Conclusions

There are various guidelines in the literature on MG management. Physicians usually adhere to and achieve confidence and familiarity with specific treatment plans. However, the “recipe” for treatment can and should be designed for the individual patient's comorbidities. The aim in MG treatment is to induce remission or MM and to enable patients to resume their normal life-style. Each patient, however, is unique with respect to their comorbidities and their social or personal circumstances. As a result, the immunosuppressive therapy prescribed needs to be “catered” for that particular individual bearing all those pertinent variables in mind. Residual myasthenic symptoms, which physicians may perceive as minimal may have a significant impact on a patient's daily life. As physicians, we need to be mindful of the impact of patients' MG on their physical and mental health, the impact on their family or carers, and the impact of adverse effects from MG-related therapies on their general health. The development of new therapies for the severe end of the MG spectrum is exciting. We need to learn more about these drugs, gain familiarity and identify the patient groups who are more likely to benefit from them. Detailed cost-utility analysis is required for individual health-care systems to enable physicians in their process of justifying the use of these drugs to their respective hospital systems. Addressing fatigue and its management is paramount to the overall MG management. Encouraging patients to exercise should be an integral part of their treatment since this will help their overall well-being in the long-term. Finally, dysfunctional breathing should be recognized and treated accordingly.

## Author Contributions

MF and JG contributed equally to conceptualizing this document. MF wrote the manuscript with contributions from JG to different sections. All authors contributed to the article and approved the submitted version.

## Conflict of Interest

The authors declare that the research was conducted in the absence of any commercial or financial relationships that could be construed as a potential conflict of interest.

## Abbreviations

AChR: Acetylcholine receptor
C5: Complement component 5
CSR: complete stable remission
DM1: myotonic dystrophy type 1
EFT: early fast-acting treatment
FSHD: Facioscapulohumeral muscular dystrophy
IvIG: intravenous immunoglobulin
MG: Myasthenia gravis
MGFA: Myasthenia Gravis Foundation of America
MGTX: thymectomy trial in non-thymomatous MG patients
MM: minimal manifestations
MMF: mycophenolate mofetil
MuSK: Muscle specific tyrosine kinase
NIV: non-invasive ventilation
PE: plasma exchange/plasmapheresis
PIS: post-intervention status
PR: pharmacological remission
RATS: robotic assisted thymectomy surgery
VATS: Video-assisted thoracoscopic surgery.
